# The association between timing of dietary macronutrient and sodium consumption and sleep duration and quality

**DOI:** 10.1093/sleepadvances/zpae007

**Published:** 2024-01-23

**Authors:** Velarie Yaa Ansu Baidoo, Shaina J Alexandria, Phyllis C Zee, Kristen L Knutson

**Affiliations:** Postdoctoral fellow, Center for Circadian and Sleep Medicine, Department of Neurology, Northwestern University Feinberg School of Medicine, Chicago, IL, USA; Department of Preventive Medicine, Northwestern University Feinberg School of Medicine, Chicago IL, USA; Center for Circadian and Sleep Medicine, Department of Neurology, Northwestern University Feinberg School of Medicine, Chicago, IL, USA; Center for Circadian and Sleep Medicine, Department of Neurology, Northwestern University Feinberg School of Medicine, Chicago, IL, USA

**Keywords:** sleep, macronutrients, sodium, wake after sleep onset, polysomnography, sleep quality, sleep duration, circadian rhythm

## Abstract

**Study Objective:**

The objective of this study was to examine the association between the timing of dietary macronutrients and sodium intake and sleep quantity and quality.

**Methods:**

This was a cross-sectional study that included 34 adults between 21 and 50 years of age. The main outcome measures were objective sleep measures assessed from three nights of wrist actigraphy including sleep duration, fragmentation, and wake after sleep onset (WASO), and one night of polysomnography (PSG), including rapid eye movement (REM) sleep, non-REM stage 2 (N2), stage 3 (N3), and WASO. Multiple linear regression models and linear mixed models were used to estimate the associations between sleep measures and dietary measures (carbohydrates, fats, saturated fats, proteins, and sodium). Dietary timing was examined in two ways: (1) the average amount of each nutrient consumed within 3 hours of sleep start, and (2) the interval between the final intake of each nutrient and sleep.

**Results:**

Average fat intake within 3 hours of sleep was associated with greater WASO from PSG (*β* = 4.48, *p* = 0.01). No other associations were found between the macronutrients or sodium intake (*p* > 0.05) within 3 hours of sleep and the sleep parameters from PSG or actigraphy. Similarly, no associations were found between any of the PSG or actigraphy sleep measures and the interval between final nutrient intakes and sleep with sleep duration.

**Conclusions:**

The study suggests that greater fat but not carbohydrate, protein, saturated fat, or sodium intake close to sleep may be associated with greater sleep disruption; however, no other associations were observed.

**Statement of Significance**

This study adds to the literature on the association between dietary intake and sleep quality. Findings from this observational study show that intake of a high-fat meal or snack close to sleep (within 3 hours) was associated with poorer sleep quality, specifically greater wake after sleep onset. An important aspect of this study is the use of a 3-day food diary to assess meal/snack intake and the use of two objective sleep assessment methods: polysomnography and wrist actigraphy.

## Introduction

Sleep is essential for promoting the health of individuals. Short sleep duration and poor sleep quality have been associated with adverse health consequences such as cardiovascular disease, obesity, and type 2 diabetes [1–3]. In addition, many physiological functions, including sleep, are regulated by the circadian system, which in turn is influenced by behaviors such as eating. Thus, inappropriate meal timing, particularly relative to sleep timing, may result in a misalignment in the internal circadian clock and could adversely affect sleep.

Some prior research has linked nutrient intake to sleep. Long-term studies have shown that diets higher in carbohydrates positively affect rapid eye movement (REM) sleep but negatively affect non-REM sleep. Also, high protein diets above recommendations were shown to affect sleep negatively while inconclusive results were obtained for fat intake [4]. On the other hand, inconclusive results have been found for acute (interventions <24 hours) macronutrient (protein and fat) manipulation studies [4]. The timing of these macronutrients was not included in the studies and may in part account for the inconsistent effects. Results from a systematic review of 11 clinical trials showed that consuming a low carbohydrate (vs. high carbohydrate) diet moderately increased sleep duration [5].

The Western diet is characterized by a high intake of sodium with Americans consuming an average of more than 3400 mg/d [6] as compared to the upper limit recommendation of 2300 mg/d. A small amount of sodium is essential for proper body functioning; however, excessive intake increases the risk of cardiovascular diseases, blood pressure, and sleep disturbances [7]. Consuming foods high in dietary sodium has been linked to high blood pressure and mortality [8]. One study (*n* = 97) found that dietary sodium was associated with the severity of obstructive sleep apnea among patients with resistant hypertension [9]. In another study, a randomized controlled trial (*n* = 54) reduced salt and water content in the body, which led to a reduced apnea–hypopnea index (which indicates the severity of sleep apnea) in men diagnosed with severe obstructive sleep apnea [10]. Whether sodium is linked to other indicators of sleep quality beyond sleep apnea is unknown.

Understanding how the timing, amount, and composition of dietary intake affect sleep health will provide useful insight into possible strategies to improve sleep, which in turn could help to improve overall well-being. This study aimed to explore the association between the timing and amount of intake of dietary macronutrients and sodium on sleep quantity and quality. As such, we have conceptualized “timing” and “amount” in two ways. We examined the amount of each nutrient consumed within 3 hours of sleep start and hypothesized that consuming more macronutrients and sodium close to sleep (within 3 hours) would be associated with shorter sleep duration and lower sleep quality. Second, we examined meal timing relative to sleep by calculating the interval between final intake and sleep start and hypothesized that consuming macronutrients and sodium closer to sleep start (i.e. a shorter interval) would be associated with shorter sleep duration and lower sleep quality.

## Materials and Methods

### Study participants and design

The cross-sectional study enrolled volunteers who identified as black/African Americans or non-Hispanic white and were between the ages of 21 and 50 years. Exclusion criteria included: body mass index (BMI) ≥ 40 kg/m^2^, diabetes, sleep disorders, medication use (excluding lipid-lowering drugs and antihypertensive medications), shift workers, history of Lasik eye surgery, or color blindness. Sleep disorders were identified based on self-reported existing sleep disorders, an overnight full PSG recording in the laboratory, and physician interviews. The overall goal of the parent study (*n* = 44) was to examine associations between sleep and circadian markers and glucose tolerance and whether the associations varied by race. The study was conducted at the University of Chicago between April 2013 and November 2016 [11]. The University of Chicago, Illinois institutional review board approved the study protocol, and all study participants provided written informed consent before participating.

### Sleep assessment

Measures of sleep were assessed in study participants’ homes and included two devices: wrist actigraphy and polysomnography (PSG). PSG is the gold standard for assessing sleep stages, but it is cumbersome and only collected for one night. Actigraphy, on the other hand, can be collected for multiple days to provide more habitual estimates. Participants were instructed to wear waterproof wrist actigraphy monitors (Actiwatch-2 or Actiwatch Spectrum, Respironics/Philips, Bend, OR) on their non-dominant hand for 10 days to estimate habitual sleep metrics, including sleep duration, sleep start time, wake after sleep onset (WASO), and sleep fragmentation (i.e. repeated short sleep interruptions). For each night that the actigraphy monitors were worn, the participants were asked to press the event marker on the actigraph (which does not interrupt data recording) whenever they tried to sleep and when they woke up in the morning. Additionally, participants were instructed to record their wake and sleep start times in a sleep diary on the days that the wrist actigraphy was worn. The sleep diary served as a backup for sleep start and end times when the participant did not press the event marker and as a form of data validation. Actigraphy was scored using the Actiware software. For the present analyses, we averaged only the 3 days that corresponded to the food diary days.

One night of unattended, at-home PSG recording was performed as PSG is the only validated method to assess sleep stages. The PSG used an ambulatory digital acquisition system (Track-it, Nihon Kohden, CA). Recordings included electroencephalography (EEG), electrooculography (EOG), electromyography (EMG), and electrocardiography (ECG) signals. Each 30-second epoch of recording was staged as either wake, non-rapid-eye-movement stage 1 (N1), stage 2 (N2), stage 3 (N3; also known as slow-wave sleep), or REM following standard criteria [12, 13]. Sleep stage raters were experienced and blinded to the age, gender, and race of the volunteer. For these analyses of PSG outcomes, participants with total sleep times on the PSG of <4 hours were excluded (*n* = 6).

### Dietary assessment

A consecutive 3-day food diary was completed by participants. Fifteen participants completed the diary on Thursday, Friday, and Saturday; 16 completed it on Wednesday, Thursday, and Friday; two completed it on Saturday, Sunday, and Monday; and one completed it on Sunday, Monday, and Tuesday. The 3 days of food records were matched with the 3 days when the wrist actigraphy monitors were worn. Therefore, only food diaries collected simultaneously with actigraphy monitors were included in these analyses. Additionally, PSG was recorded during this week, but not on the same day as food diary records (to minimize participant impacts). Foods were reported by study participants as either breakfast, lunch, dinner, or snack. Study participants were provided with detailed instructions on how to record a food diary and all completed food records were reviewed by a trained professional to ensure completeness. Meals were defined as an eating event where energy intake was <5 kcal, and timing between meals was at least 15 minutes. Dietary intake data were analyzed using Food Processor Nutrition Analysis Software ESHA (version 10.7.0, Salem, OR) [14] which was used to calculate carbohydrates, protein, fat (in grams), saturated fat (in grams), and sodium (in micrograms) in each meal/snack, including the final meal/snack of the day.

For each nutrient, we calculated two metrics to assess nutrient consumption close to sleep. For each day with both actigraphy and dietary intake data, we calculated the interval between the timing of the final intake of each nutrient and sleep start time from actigraphy. This was done separately for each nutrient because not all final meals/snacks included all nutrients. Our second metric involved summing the amount of each nutrient consumed in the 3 hours prior to sleep start for each diary day. For both metrics, we averaged the three daily values. Three hours close to sleep time was chosen based on existing literature [15] and the common sleep hygiene recommendation [16, 17] of avoiding meals and beverages close to sleep since it presumably interrupts sleep.

### Covariates

Participants self-reported their age, gender (male or female), and race (African American or white). Height and weight were measured by study personnel and BMI was calculated (kg/m^2^).

### Statistical analyses

Descriptive results of the study characteristics were summarized as means ± standard deviations for continuous variables and as percentages for categorical variables. We performed two sets of analyses and separate models were fit for each nutrient (carbohydrates, proteins, fat, saturated fat, and sodium). The first set of analyses estimated the association between the average amount of each nutrient consumed within 3 hours of sleep start and the sleep variables. Multiple linear regression models were used to estimate the association between the 3-day averages for all nutrients consumed within 3 hours and the PSG sleep measures. For the actigraphy outcomes, we used linear mixed models since we have multiple days of actigraphy and food intake measurements. These models included a random intercept for participant and compound symmetry covariance structure and also adjusted for the participant’s BMI, sleep start time, and whether any nutrients were consumed within 3 hours of sleep start. The second set of analyses analyzed the interval between the time of the final intake of each nutrient and the time of sleep start, adjusted for BMI, sleep start time, and the amount of nutrients in the last meal or snack. Similar to the first set of analyses, multiple linear regression models were used to estimate the association between the 3-day averages of these intervals and the PSG sleep measures and mixed models were used for the actigraphy sleep measures. All statistical analyses were performed using SAS version 9.4 (SAS Institute Inc., Cary, NC, USA), and a *p*-value *p* < 0.05 was considered statistical significance.

## Results

### Descriptive and multivariable model results

Results from the demographic, dietary, and sleep characteristics of the study sample are presented in Table 1. Thirty-four participants (17 men and 17 women) ages 21 to 49 years met the inclusion criteria for this study. The majority of the study participants were white adults (65%). The average amount of carbohydrates, proteins, fat, saturated fat, and sodium are provided in the table. Thirteen (38%) participants did not consume any nutrients during this 3-hour window. The average interval between last nutrient consumption and sleep start time was 4.2–4.4 hours and ranged from 1.0 to 9.0 hours. Study participants slept for about 7 hours on average during the PSG and had an average of 77 minutes of N3 sleep, 112 minutes of REM sleep, and 45 minutes of WASO. The average sleep duration from actigraphy was 407 minutes (6.8 hours), sleep fragmentation averaged 17% and there was an average of 39 minutes of WASO from actigraphy.

**Table 1.** Demographic and Sleep Characteristics of Adult Volunteers in the Study (*n* = 34)

	Mean (SD) or *N* (%)
Demographics	
Age (years)	30.2 (7.5)
Body mass index (kg/m^2^)	26.8 (5.2)
*Gender*	
Male	17 (50%)
Female	17 (50%)
Race	
African American	11 (35.3%)
White	23 (64.7%)
*Average amount consumed within 3 hours of bedtime*	
Carbohydrates (g)	21.6 (26.8)
Proteins (g)	6.2 (8.4)
Fat (g)	7.0 (8.2)
Sodium (mg)	294.2 (590.7)
Saturated fat (g)	2.4 (3.3)
Calories (kcal)	188.9 (235.3)
*Interval between final consumption of each nutrient and bedtime*	
Time of last meal/snack (hours)	20.36 (1.11)
Carbohydrate-sleep interval (hours)	4.2 (1.5)
Protein-sleep interval (hours)	4.4 (1.9)
Fat-sleep interval (hours)	4.3 (1.7)
*Amount in last meal/snack before bedtime*	
Carbohydrate (g)	73.5 (34.9)
Protein (g)	27.9 (19.4)
Fat (g)	25.5 (16.7)
*PSG sleep measures*	
Total sleep time (hours)	7.2 (1.2)
Stage N2 sleep (minutes)	219.31 (51.32)
Stage N3 sleep (minutes)	76.9 (25.6)
Stage REM sleep (minutes)	110.9 (39.5)
WASO (minutes)	46.9 (62.9)
*Actigraphy sleep measures*	
Sleep start time (hour)	24.31 (1.11)
Sleep duration (minutes)	408.6 (60.5)
Sleep fragmentation (%)	17.4 (5.9)
WASO (minutes)	38.6 (16.4)

PSG, polysomnography; WASO, wake after sleep onset; REM, rapid eye movement.

#### Consumption within 3 hours of sleep starts.

The results from the regression analyses (Tables 2–5) are presented as adjusted estimated *β*-values with corresponding *p*-values, including the overall *p*-value for each model. We first examined associations between energy and nutrient intake within 3 hours of sleep start and PSG sleep measures (Table 2) and actigraphy sleep measures (Table 3). The multiple linear regression results showed that greater average fat intake within 3 hours of sleep was associated with greater WASO from PSG (*β* = 4.48, *p* = 0.01), which means that every additional gram of fat consumed was associated with approximately 4.5 more minutes of WASO during PSG. No significant relationships were found between the other macronutrients or sodium intake (*p* > 0.05) within 3 hours of sleep start and the other sleep parameters from PSG or actigraphy.

**Table 2.** Multiple Regression Models^a^ of the Association Between PSG Sleep Measures and Nutrient Intake

	N2 (minutes)		N3 (minutes)		REM (minutes)		WASO (minutes)	
Variable	Adjusted Estimate (SE)	*P*-value	Adjusted Estimate (SE)	*P*-value	Adjusted Estimate (SE)	*P*-value	Adjusted Estimate (SE)	*P*-value
Carbohydrates within 3 hours of sleep (g)	−0.27 (0.46)	0.56	0.01 (0.22)	0.96	−0.24 (0.35)	0.50	0.95 (0.52)	0.08
*Overall model*		0.71		0.56		0.89		0.26
Proteins within 3 hours of [1] sleep (g)	0.31 (1.41)	0.83	-0.03 (0.67)	0.96	0.09 (1.07)	0.93	2.35 (1.62)	0.16
*Overall model*		0.77		0.56		0.95		0.39
Fats within 3 hours of sleep (g)	−0.26 (1.59)	0.87	−0.42 (0.75)	0.58	−0.18 (1.21)	0.88	4.48 (1.70)	0.01
*Overall model*		0.77		0.51		0.95		0.08
Sodium within 3 hours of sleep (mg)	0.01 (0.02)	0.50	0.01(0.01)	0.39	0.004 (0.01)	0.78	0.005 (0.02)	0.83
*Overall model*		0.69		0.44		0.94		0.72
Saturated fats within 3 hours of sleep (mg)	−0.92 (3.70)	0.81	−0.40 (1.76)	0.82	−0.79 (2.83)	0.78	−8.27 (4.13)	0.05
*Overall model*		0.77		0.55		0.94		0.21

PSG, polysomnography; REM, rapid eye movement; WASO, wake after sleep onset.

^a^Each model is adjusted for BMI, sleep start time, and whether nutrient was consumed within 3 hours (*n* = 34).

**Table 3.** Mixed Model Analysis of the Association Between Actigraphy Sleep Measures with Macronutrient and Sodium

	Sleep duration (minutes)		Fragmentation (minutes)		WASO (minutes)	
Variable	Adjusted estimate (SE)	*P*-value	Adjusted estimate (SE)	*P*-value	Adjusted estimate (SE)	*P*-value
Carbohydrates within 3 hours of sleep (g)	−0.08 (0.30)	0.80	−0.02 (0.03)	0.53	0.05 (0.07)	0.54
*Overall model*		0.001		0.86		0.09
Proteins within 3 hours of sleep (g)	−0.29 (0.67)	0.67	−0.04 (0.06)	0.49	0.09 (0.16)	0.61
*Overall model*		0.0001		0.89		0.08
Fats within 3 hours of sleep (g)	0.11 (0.65)	0.86	0.002 (0.06)	0.98	0.12 (0.16)	0.46
*Overall model*		0.001		0.90		0.08
Sodium within 3 hours of sleep (mg)	−0.001(0.01)	0.91	0.0002 (0.001)	0.92	0.001 (0.003)	0.63
*Overall model*		0.0001		0.87		0.09
Saturated fats within 3 hours of sleep (g)	0.07 (1.59)	0.96	−0.03 (0.14)	0.85	0.24 (0.39)	0.54
*Overall model*		0.0001		0.91		0.07

WASO, wake after sleep onset.

^a^Each model is adjusted for BMI, sleep start time, and whether nutrient was consumed within 3 hours (*n* = 34).

#### The interval between last meal/snack and sleep start.

We examined whether the duration between the last meal/snack and sleep start was associated with sleep measures based on PSG (Table 4) and actigraphy (Table 5). No significant associations were found between the interval between nutrient intakes and sleep start and any of the PSG sleep measures.

**Table 4.** Multiple Regression Model^a^ of the Association Between PSG Sleep Measures and Timing of Macronutrient Intake

	N2 (minutes)		N3 (minutes)		REM (minutes)		WASO (minutes)	
Variable	Adjusted estimate (SE)	*P*-value	Adjusted estimate (SE)	*P*-value	Adjusted estimate (SE)	*P*-value	Adjusted estimate (SE)	*P*-value
Carbohydrate-sleep interval (hr.)	11.11 (8.16)	0.18	−3.54 (4.04)	0.39	−6.66 (6.19)	0.29	6.72 (9.12)	0.47
* Overall model*		0.46		0.74		0.57		0.11
Protein-sleep interval (hr.)	0.31 (5.65)	0.96	1.46(2.79)	0.61	6.60(4.02)	0.11	4.81(6.68)	0.48
* Overall model*		0.98		0.59		0.59		0.38
Fat-sleep interval (hr.)	9.15 (7.5)	0.23	−1.26(3.75)	0.74	−7.44 (5.71)	0.20	4.63 (2.31)	0.61
*Overall model*		0.54		0.84		0.70		0.44

PSG, polysomnography; REM, rapid eye movement; WASO, wake after sleep onset.

^a^Each model is adjusted for BMI, sleep start time, (*n* = 34) and macronutrient amount in meal/snack.

**Table 5.** Mixed Model Analysis^a^ of the Association Between Actigraphy Sleep Measures and Timing of Macronutrient Intake

	Sleep duration (minutes)		Fragmentation (minutes)		WASO (minutes)	
Variable	Adjusted estimate (SE)	*P*-value	Adjusted estimate (SE)	*P*-value	Adjusted estimate (SE)	*P*-value
Carbohydrate-sleep interval (hr.)	−7.85 (5.55)	0.16	0.46 (0.47)	0.34	0.81 (1.35)	0.55
*Overall model*		0.001		0.58		0.08
Protein-sleep interval (hr.)	−7.06 (4.72)	0.14	−0.07 (0.04)	0.56	−0.23 (1.15)	0.84
*Overall model*		0.001		0.94		0.16
Fat-sleep interval (hr.)	−9.27 (5.28)	0.08	0.33(0.44)	0.46	−0.01(0.08)	0.51
*Overall model*		0.001		0.62		0.07

WASO, wake after sleep onset.

^a^Each model adjusted for BMI, sleep start time, (*n* = 34), and macronutrient amount in meal/snack.

## Discussion

This study examined whether macronutrients (carbohydrates, proteins, fats, and saturated fats) or sodium intake close to bedtime were associated with sleep quantity or quality. We assessed timing close to sleep start in two different ways: (1) by estimating the amount consumed within a 3-hour period prior to sleep start and (2) by calculating the interval between final consumption and sleep start. Findings from this observational study suggest that eating foods high in fats close to sleep may be associated with a greater wake after falling asleep (WASO) based on PSG. However, no actigraphy measure nor any other PSG measure was associated with these nutrient measures.

Other studies have examined associations between sleep and nutrient intake. For example, Crispim et al. also observed an association between greater WASO and greater fat intake (*β* = 0.55, *p* = 0.01) and found no significant association between each of the macronutrients (carbohydrates, proteins, and fats) and N3% (*p* > 0.05) [18], which is consistent with the current study findings. Other studies found a relationship between carbohydrate intake and WASO, which is contrary to our findings. A study among female athletes (*n* = 32) found that greater daily carbohydrate intake was associated with greater WASO (*β* = 0.05, *p* = 0.01), which is contrary to the present study findings. However, similar to our findings, that study did not observe a significant relationship between WASO and protein (*β* = −0.04, *p* = 0.19) or sodium (*β* = 0.002, *p* = 0.15) intake [19]. Results from a cross-sectional survey study of 793 college students did find a significant association between eating <3 hours before bedtime and more reported nocturnal awakening (OR = 1.61, 95% CI = 1.15 to 2.27) but no association with short self-reported sleep duration [15]. Additional larger studies are warranted to examine the timing of macronutrient intake utilizing validated instruments to confirm associations between diet and sleep.

When we examined the association between the interval between the last meal/snack and sleep start and the sleep measures, no significant relationships were found. Other studies, however, have found associations between the timing of food intake and measures of sleep. For example, the American Time Use Survey [17] showed that participants (*n* = 124 239) who ate close (<1 hour) to bedtime were more likely to self-report greater WASO (≥ 30 minutes) (OR = 2.26, 95% CI: 1.93,2.64) as compared to those who did not eat close to bedtime (<2 hours) (OR = 2.01, 95 % CI: 1.80, 2.25) or < 3 hours (OR = 1.80, CI: 1.63, 1.99) [17]. Participants in the ATUS study [17] only recorded whether they had eaten/drunk anything within specific time intervals but they did not record specific meals, hence specific nutrients could not be assessed. Similar to our findings, results from a meta-analysis of nine cross-sectional cohort studies (*n* = 14 906) [20] found no associations between sleep duration and carbohydrate (*β* = −0.07, *p* = 0.23), fat (*β* = 0.02, *p* = 0.99), and protein (*β* = 0.001, *p* = 0.96) habitual intake. Of note, sleep duration was self-reported and dietary intake was obtained from FFQ in the above cohort study [20]. Also, the ATUS [21] study discussed above showed that mealtimes less than an hour before bedtime during the weekday were associated with long sleep duration (>9 hours; OR = 1.79, 95% CI: 1.67, 1.91) [21]. Overall, our findings and those from other studies were not consistent and therefore more research is required to identify whether nutrient consumption within a specific window of time prior to sleep can impair sleep quality.

There are several potential mechanisms to explain an association between nutrient intake close to bedtime and poorer sleep quality. One such mechanism could be due to circadian disruption. The circadian system regulates sleep propensity, and the circadian system is influenced by behaviors such as eating. In fact, meal intake is an important synchronizing signal for many—but not all—of the tissues’ circadian clocks. Thus, when food is consumed at an inappropriate circadian time, according to the central clock, there could be internal circadian disruption, which may impair the circadian regulation of sleep and sleep quality [20]. In addition, inappropriate meal timing may lead to inefficient digestion and nutrient metabolization [22], could lead to impaired sleep if it leads to discomfort. The second potential mechanism underlying this relationship, therefore, could be because of gastroesophageal reflux disease (GERD), or gastric issues from indigestion [23]. Fat consumption close to bedtime may result in these gastrointestinal effects, which could in turn disturb sleep and increase wake [23]. Additionally, the intake of high-fat foods slows gastric emptying and may cause a relaxation of the lower esophageal sphincter which may contribute to postprandial GERD symptoms [24, 25]. Indeed, the American College of Gastroenterology recommends avoiding meals within 2–3 hours of bedtime to avoid GERD [26]. As such, consuming foods high in fat close to bedtime should be avoided.

One strength of this current study is utilizing two objective assessments of sleep, including PSG, which is the gold standard method for measuring sleep stages, and wrist actigraphy, which can collect multiple days and capture daily variability, making it a better assessment of habitual sleep than a single night. The study also recorded the timing of meals/snacks in addition to the content in a food diary over 3 days. This study also has some limitations, including the study’s small sample size (*n* = 34), which may decrease statistical power to detect associations. Furthermore, results may not be generalizable to other age groups, ethnicities, or other regions where diets may differ. Also, dietary data are self-reported and are therefore prone to reporting errors. Additionally, due to the cross-sectional nature of the study design, no causal relationship can be inferred. Lastly, it is important to note that both diet and sleep are complex and variable, and thus future work should consider the combined effects of multiple nutrients, as well as subcategories of nutrients, on sleep.

## Conclusions

Findings from this study suggest a possible link between greater fat intake close to sleep and poor sleep quality (e.g. greater wake time). Future experimental studies should use rigorous measures to manipulate dietary intake relative to bedtime and assess sleep objectively to further understand the relationship between sleep and diet. These intervention studies could test whether manipulating diet, particularly fat intake, can improve sleep. Healthy sleep is important for general health and well-being and therefore methods to improve sleep could have broad beneficial health effects.

## Data Availability

The data for this article will be available to share on reasonable request to the corresponding author.
